# Retraction: The Combination of Three Natural Compounds Effectively Prevented Lung Carcinogenesis by Optimal Wound Healing

**DOI:** 10.1371/journal.pone.0294975

**Published:** 2023-11-21

**Authors:** The PLOS ONE Editors

Following the publication of this article [1], concerns were raised regarding Figs 2, 3, 6, 7, and 9. Specifically:

- In Fig 2B, the aconitine panel appears similar to the TIF panel found in Fig. 6B of [2]
- The following graphs appear similar despite representing different experimental conditions:
  ○ Fig 3C and Fig 3D
  ○ Fig 6C, Fig 6D, and Fig 6E
  ○ Fig 9B and Fig 9C
- In Fig 7A, there appear to be vertical discontinuities between lanes 1 and 2 in the Oct4 panel and lanes 1 and 2 in the Nanog panel.
- In Fig 7A the GAPDH panel appears similar to the beta-actin panel found in Figure 4E of [3].
- In Fig 7B, the Notoginsenoside R1 Cleaved Caspase-3 panel appears similar to the Control Pimonidazole panel in Fig. 6A of [2].
- In Fig 9A the left region of the Shikonin panel appears to overlap with the right region of the Notoginsenoside R1 panel.
- In Fig 9A the control, Shikonin, Aconitine Notoginsenoside R1 and combined three compounds panels appear similar to the correspondingly named panels in Fig. 7 of [4].

The authors stated that the underlying data for all figures was no longer available. Regarding the concerns in Fig 2B, Fig 7A and Fig 7B the authors stated they were caused by errors in arranging data in the submission process for [2] and [3] and that they had applied for corrigendum in the respective journals. The authors stated that the similar graphs found in Fig 3 and Fig 6 had similarities in trend but were not the same data, however individual level data was not made available for editorial review. The PLOS Editors remain concerned about these figures. The authors stated the vertical discontinuities in Fig 7A were caused by alterations to the background to highlight the differences between samples. The PLOS Editors remain concerned about this figure. The authors stated the similarities in the Shikonin and Notoginsenoside panels in Fig 9A were the result of errors in figure preparation.

In light of the concerns affecting multiple figure panels that question the integrity and reliability of these data, the *PLOS ONE* Editors retract this article.

All authors either did not respond directly to the editorial decision or could not be reached.
